# The type III protein secretion system contributes to *Xanthomonas citri* subsp. *citri* biofilm formation

**DOI:** 10.1186/1471-2180-14-96

**Published:** 2014-04-18

**Authors:** Tamara Zimaro, Ludivine Thomas, Claudius Marondedze, Germán G Sgro, Cecilia G Garofalo, Florencia A Ficarra, Chris Gehring, Jorgelina Ottado, Natalia Gottig

**Affiliations:** 1 Instituto de Biología Molecular y Celular de Rosario, Consejo Nacional de Investigaciones Científicas y Técnicas (IBR-CONICET) and Facultad de Ciencias Bioquímicas y Farmacéuticas, Universidad Nacional de Rosario. Ocampo y Esmeralda, Rosario 2000, Argentina; 2 Biological and Environmental Science and Engineering Division, King Abdullah University of Science and Technology, Thuwal 23955-6900, Saudi Arabia

**Keywords:** *Xanthomonas citri* subsp. *citri*, Biofilm, T3SS, Proteomics

## Abstract

**Background:**

Several bacterial plant pathogens colonize their hosts through the secretion of effector proteins by a Type III protein secretion system (T3SS). The role of T3SS in bacterial pathogenesis is well established but whether this system is involved in multicellular processes, such as bacterial biofilm formation has not been elucidated. Here, the phytopathogen *Xanthomonas citri* subsp. *citri* (*X. citri*) was used as a model to gain further insights about the role of the T3SS in biofilm formation.

**Results:**

The capacity of biofilm formation of different *X. citri* T3SS mutants was compared to the wild type strain and it was observed that this secretion system was necessary for this process. Moreover, the T3SS mutants adhered proficiently to leaf surfaces but were impaired in leaf-associated growth. A proteomic study of biofilm cells showed that the lack of the T3SS causes changes in the expression of proteins involved in metabolic processes, energy generation, exopolysaccharide (EPS) production and bacterial motility as well as outer membrane proteins. Furthermore, EPS production and bacterial motility were also altered in the T3SS mutants.

**Conclusions:**

Our results indicate a novel role for T3SS in *X. citri* in the modulation of biofilm formation. Since this process increases *X. citri* virulence, this study reveals new functions of T3SS in pathogenesis.

## Background

The bacterial genus *Xanthomonas* comprises a number of Gram-negative plant pathogenic bacteria that cause a variety of severe plant diseases [1]. *Xanthomonas citri* subsp. *citri*, the phytopathogen causing citrus canker, invades host plant tissues entering through stomata or wounds and then colonizes the apoplast of fruit, foliage and young stems, causing raised corky lesions and finally breaking the epidermis tissue due to cell hyperplasia, thus allowing bacterial dispersal to other plants [2].

Persistent and severe disease can lead to defoliation, dieback and fruit drop, reducing yields and causing serious economic losses [3]. To date, no commercial citrus cultivars are resistant to citrus canker and current control methods are insufficient to manage the disease [3]. Thus, there is a need to study the infection process in order to enable the development of new tools for disease control. Furthermore, the study of *X. citri*-citrus interactions has been used as a model to provide new advances in the understanding of plant-pathogen interactions [1].

The Type III protein secretion system (T3SS) is conserved in many Gram-negative plant and animal pathogenic bacteria [4]. The T3SS is subdivided into (i) the non-flagellar T3SS (T3aS) involved in the assembly of the injectisome or hypersensitive response and pathogenicity (Hrp) pilus, and (ii) the flagellar T3SS (T3bS), responsible for assembly of the flagellum [5]. The T3SS spans both bacterial membranes and is associated with an extracellular filamentous appendage, termed ‘needle’ in animal pathogens and ‘Hrp pilus’ in plant pathogens, which is predicted to function as a protein transport channel to the host-pathogen interface [4]. Translocation of effector proteins across the host membrane requires the presence of the T3SS translocon, a predicted protein channel that consists of bacterial Type III-secreted proteins [6].

A number of surface appendages, such as conjugative pili, flagella, curli, and adhesins have been shown to play a role in biofilm formation [7,8]. The role of T3SS as an effector protein delivery machine is well established, however, whether this secretion system participates in multicellular processes such as biofilm formation remains unanswered. Several studies concluded that T3SS is only necessary for pathogenicity and that expression of this secretion system is repressed in biofilm-growing bacteria. For example, *Pseudomonas aeruginosa* PA14 *sadRS* mutant strains that cannot form biofilms have enhanced expression of T3SS genes, while a *P. aeruginosa* PA14 T3SS mutant exhibits enhanced biofilm formation compared to wild type strain [9]. Furthermore, in *Yersinia pseudotuberculosis*, it has been shown that the T3SS needle blocks biofilm formation in the model host *Caenorhabditis elegans*[10]. In contrast, other studies highlighted the role of T3SS in bacterial biofilm formation. Microarray experiments performed in *P. aeruginosa* cystic fibrosis epidemic strain AES-2 showed expression of T3SS encoding genes up-regulated in biofilms as compared to planktonic bacteria [11]. In the plant pathogen *Erwinia chrysanthemi*, it has been shown that the T3SS pilus is involved in the aggregative multicellular behavior that leads to pellicle formation [12]. The enterohemorrhagic *Escherichia coli* O157 has a well-defined T3SS, termed *E. coli* Type III secretion system 1 (ETT1), which is involved in attachment and effacement and is critical for virulence. This strain also has a gene cluster potentially encoding an additional T3SS (ETT2) [13]. Studies on an ETT2 deletion mutant strain showed that although ETT2 is not responsible for protein secretion, it is involved in biofilm formation and hence in virulence [13]. Recently, it has been shown that the *Salmonella enterica* serovar *Typhimurium* T3SS secretion system SPI-1 is involved in the formation of an adherent biofilm and cell clumps in the culture media [14]. Taken together, the evidence suggests that T3SS may play a role in bacterial biofilm formation.

In *X. citri*, biofilm formation is required for optimal virulence as revealed by several reports with different bacterial mutants. For instance, *X. citri* mutants that are unable to biosynthesize molecules needed for biofilm formation such as exopolysaccharide (EPS), an adhesin protein and the lipopolysaccharide show a reduced virulence [15-17]. Consistent with this, *X. citri* infection is reduced by foliar application of compounds that are able to inhibit *X. citri* biofilm formation [18]. The role of *X. citri* T3SS in pathogenicity is well known since T3SS mutants are unable to grow in host plants indicating that *X. citri* T3SS is responsible for the secretion of effector proteins [19]. Taking into account that biofilm formation is a requirement for *X. citri* to achieve full virulence, we have characterized the ability of a T3SS mutant to form biofilms and by performing a proteomic analysis we have identified differentially expressed proteins with a view to obtain a greater understanding of this process.

## Results

### The T3SS contributes to *X. citri in vitro* biofilm formation

**Figure 1:**
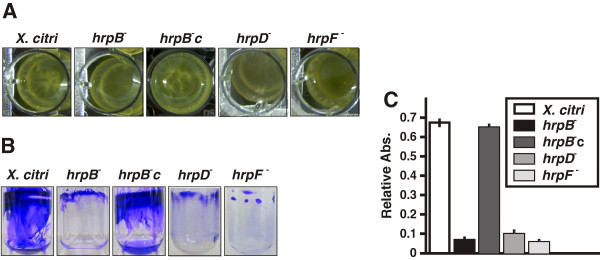
**Biofilm assays for *X. citri*, the *hrp *mutants and the *hrpB***^**−**^**c strain.** Representative photographs of biofilm formation assays for *X. citri*, *hrp* mutants and *hrpB*^−^c strains grown statically in 24-well PVC plates **(A)** or in borosilicate glass tubes **(B)** for seven days in XVM2 medium. **(C)** Quantification of biofilm formation by CV stain measured spectrophotometrically (Abs. at 600 nm). Relative Abs. indicates: CV Abs. 600 nm/Planktonic cells Abs. 600 nm. Values represent the mean from seven tubes for each strain. Error bars indicate the standard deviation.

**Figure 2:**
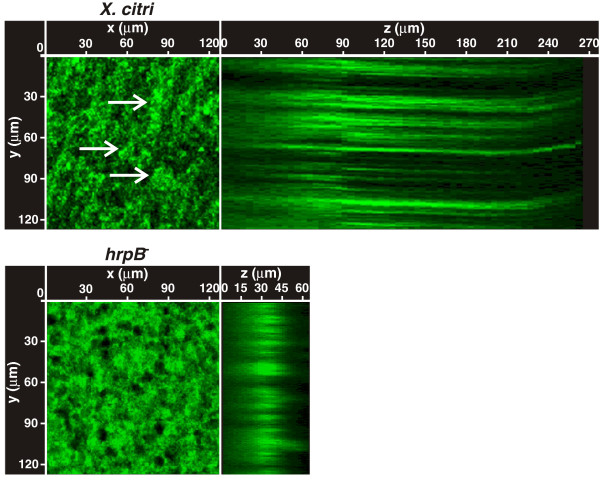
**Confocal laser scanning microscopy analysis of *X. citri *and *hrpB***^**− **^**strains grown statically.** GFP-expressing *X. citri* and *hrpB*^−^ strains cultured statically *in vitro* were analyzed by confocal laser scanning microscopy, serial images were taken at 0.5 μm distances (vertical z-stack). Z represents the ZX axis projected images. At the XY images, white arrows point to *X. citri* clusters of aggregated cells.

In order to study the role of the T3SS in *X. citri* biofilm formation, a *X. citri* T3SS mutant in the *hrpB* operon termed *hrpB*^−^ mutant [19] was characterized in their ability to form a biofilm compared to the wild type strain. The *hrpB*^*−*^ mutant was previously obtained by single crossover plasmid integration in the region that comprises the 3′ end of hrpB5 and the 5′ region of the ATPase *hrcN*[19] (Additional file 1: Figure S1A). Here, in order to complement this strain, *hrpB5* and *hrcN* were cloned in frame in the expression plasmid pBBR1MCS-5 under the control of the *lacZ* promoter [20]. However, the resulting strain did not restore biofilm formation or pathogenicity (data not shown) suggesting that downstream genes of the *hrpB* operon, *hrpB7* and *hrcT*, may be also affected in the *hrpB*^*−*^ mutant due to polarity effects (Additional file 1: Figure S1A). Therefore, the entire region containing *hrpB5*, *hrcN*, *hrpB7* and *hrcT* was cloned in the pBBR1MCS-5 vector (Additional file 1: Figure S1A) and the resulting strain (*hrpB*^*−*^c) was tested for its ability to trigger HR in non-host plants and disease in citrus leaves (Additional file 1: Figure S1B and S1C). As expected, the HR response in non-host plants was similar for the *hrpB*^*−*^c strain and *X. citri* (Additional file 1: Figure S1B). In host tissue infections, the *hrpB*^*−*^c strain did cause lesions, though it was less virulent than *X. citri*, showing a reduction in water soaking and in canker lesion formation (Additional file 1: Figure S1C). A partial complementation was also observed by RT-qPCR assays of *CsLOB1*. This gene encodes a protein that is a member of the Lateral Organ Boundaries (LOB) gene family of transcription factors whose expression is induced by the *X. citri* TAL effector protein PthA4 [21,22]. As expected, in leaves infected with *X. citri,* an induction of *CsLOB1* was observed, the *hrpB*^*−*^ mutant did not induce the expression of this gene suggesting that this mutant is not secreting PthA4 and the *hrpB*^*−*^c strain induced *CsLOB1* expression albeit at lower levels than *X. citri* probably due a lower amount of PthA4 secreted by this strain (Additional file 1: Figure S1D). Given of the possibility that bacteria may be loosing the plasmids during the host plant assays, bacteria were extracted from plant tissues and quantified at different times using appropriate antibiotics and no loss of plasmid was observed even 30 days after infiltrations (data not shown). Therefore, this partial complementation may be due to the fact that these genes are expressed under the *lacZ* promoter and that expression levels are likely to be somewhat different from those of the endogenous genes. This proposition is supported by recent work that shows that *lac* promoter-driven expression of *hrpB1* only partially complemented the *hrpB1* mutant phenotype in susceptible plants, while complete complementation was observed for HR in pathogen resistant plants [23]. For the biofilms assays, first the strains were cultured statically in 24-well PVC plates in XVM2. After seven days of growth, *X. citri* and *hrpB*^−^c strain were able to form mature biofilms with a conformation similar of that previously observed for *X. citri* strain [16], while the *hrpB*^−^ mutant showed impaired biofilm formation (Figure 1A). Next, the strains were grown statically in borosilicate glass tubes in XVM2 medium for seven days. Staining of bacterial cells with the specific crystal violet (CV) stain showed that under these conditions *X. citri* and *hrpB*^−^c strain can form biofilms of cells that adhere to the glass surface forming a thick ring at the air–liquid surface–interface of the culture medium, whereas the *hrpB*^−^ mutant was altered in its ability to form such a structure. Instead, the *hrpB*^−^ mutant formed only a narrow ring of cells (Figure 1B). CV staining of *X. citri* and *hrpB*^−^c strains was over nine times greater than that of the *hrpB*^−^ mutant (p < 0.05) (Figure 1C), thereby confirming a reduction in the capacity of biofilm formation for the mutant. Since the *hrpB*^*−*^ mutant is a polar mutant, in order to discern whether the *hrpB5-hrcT* genes or the ‘Hrp pilus’ are involved in the process of biofilm formation, the *hrpD*^*−*^ and *hrpF*^*−*^ mutants previously obtained were analyzed [19] (Additional file 1: Figure S1A). These two mutants, like the *hrpB*^*−*^ mutant, were impaired in biofilms formation (Figure 1A, 1B and 1C). All strains showed similar growth rates in XVM2 medium under agitation, with a generation time of 200 min, indicating that mutations of *hrp* genes do not impair growth of the *hrp* mutants *in vitro* (data not shown). Further, differences in statically growing cells were analyzed by confocal laser scanning microscopy using *X. citri* and *hrpB*^*−*^ strains transformed with a pBBR1MCS-5 vector that carries a copy of the *gfp* gene (pBBR1MCS-5EGFP). The analysis showed that *X. citri* formed large clusters of aggregated cells that were not observed in the *hrpB*^−^ mutant (Figure 2). Moreover, serial images taken at 0.5 μm-distance (vertical z-stack) covering the entire well length revealed that *X. citri* formed thick bacterial biofilms of about 250 μm deep, while the *hrpB*^−^ mutant formed narrower unstructured biofilms of 50 μm in length (Figure 2).

### The T3SS is not required for attachment to host tissue but is necessary for *X. citri* biofilm formation on the leaf surface

**Figure 3:**
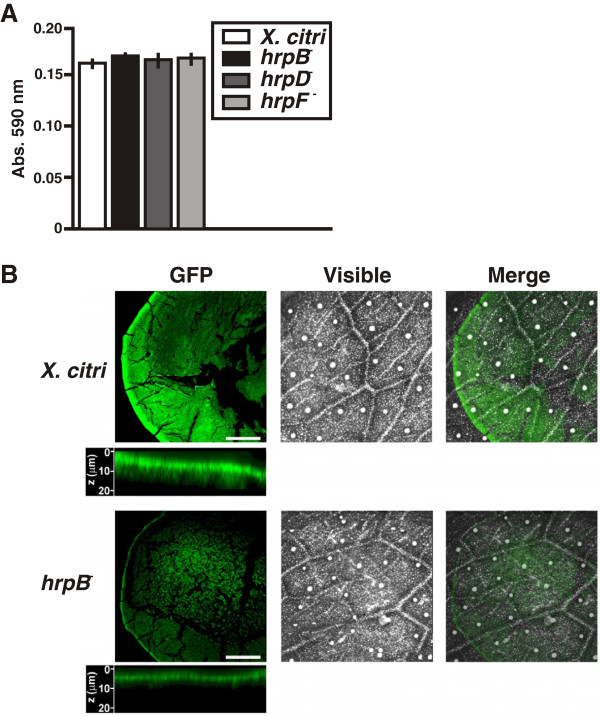
**Adherence of the *hrp *mutants to citrus leaf tissues and confocal laser scanning microscopy analysis on citrus leaves of *X. citri *and *hrpB***^**− **^**strains. (A)** Quantitative measurement of the CV retained by *X. citri* and *hrp* mutant strains adhered to abaxial leaf surfaces. Values represent the means of 20 quantified stained drops for each strain. Error bars indicate standard deviations. **(B)** Representative photographs of confocal laser scanning microscopy analysis of GFP-expressing *X. citri* and *hrpB*^−^ cells grown on leaf surfaces. Below each of the fluorescent photographs of both strains, the ZX axis projected images accumulated over serial imaging taken at 0.5 μm distances (z-stack) are shown. Scale bars: 0.5 mm.

The role of *X. citri* T3SS in bacterial adherence, like attachment to plant tissue, was evaluated by quantitative measurement of CV staining of adhered cells to leaf tissues. *X. citri* wild type and all the *hrp* mutants adhered proficiently and to the same extent to leaf tissues (p < 0.05) (Figure 3A), indicating that T3SS is not involved in leaf surface attachment. In order to analyze biofilm growth of GFP-expressing *X. citri* and *hrpB*^−^ strains on host leaf surfaces, bacterial drops were spread over the abaxial surface of citrus leaves and growth was examined confocal laser scanning microscopy. Under these conditions, *X. citri* cells grew and formed biofilm structures over the entire area of the drops on the leaf surface, with a higher density of cells accumulated at the border forming a circle (Figure 3B). The *hrpB*^−^ mutant growth was limited compared to *X. citri*, forming only small cell cumuli at the center and a narrower border circle. Further examination of the 0.5 μm stacks at the circle borders showed that *X. citri* formed a thicker bacterial biofilm of about 20 μm, while the *hrpB*^−^ mutant formed a narrower border of about 7.5 μm. These results indicate that the absence of the T3SS negatively affects biofilm formation.

### T3SS is required for *X. citri* leaf-associated survival

**Figure 4:**
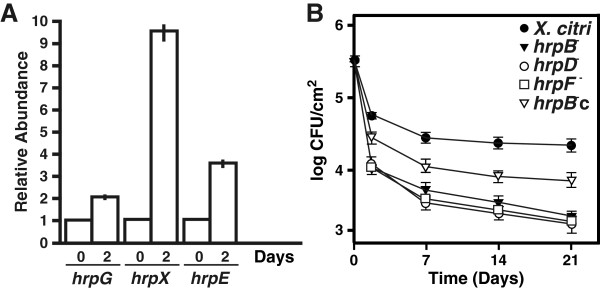
**Analysis of T3SS gene expression in leaf-associated grown *X. citri *and survival of *X. citri*, *hrp *mutants and *hrpB***^**−**^**c cells associated to leaves. (A)** RT-qPCR to determine *hrpG*, *hrpX* and *hrpE* expression levels in *X. citri* grown associated to leaves. Bars indicate the expression levels of the T3SS genes at two days of leaf-associated growth relative to time 0. Values are the means of four biological replicates with three technical replicates each. **(B)***X. citri*, *hrp* mutants and *hrpB*^−^c strains leaf-associated survival on citrus leaves. Values represent an average of four leaves assayed for each strain. Error bars indicate the standard deviation.

The expression profiles of genes involved in T3SS formation such as *hrpG* and *hrpX*, encoding for the two regulators of the *hrp* cluster [24], and *hrpE*, the major structural component of the ‘Hrp pilus’ [25] were evaluated in *X. citri* cells recovered from leaf surfaces at different times by RT-qPCR assays. A significant induction of the expression of these genes (p < 0.05) was detected after two days post-spraying of the bacteria on leaf surfaces (Figure 4A). Next, populations of the different strains were quantified at different times post-spraying on citrus leaf surfaces. One week after initial inoculation, the population size of *X. citri* decreased by almost one order of magnitude. Under these conditions, *X. citri* cannot enter through the tissue and replicate due to the thickness of the citrus leaf cuticle [16]. As a consequence, bacterial cell numbers remained relatively steady throughout the subsequent three weeks of growth. The population size of *X. citri* was nearly one order of magnitude higher at every time point analyzed (p < 0.05) as compared to the *hrp* mutants (Figure 4B). The population of the *hrpB*^−^c did not achieve *X. citri* levels, but was ever higher than that of the *hrp* mutants (Figure 4B). These results support the idea that the T3SS is necessary for *X. citri* colonization of the phyllosphere, which may be due, at least partly, to the role of T3SS in *X. citri* biofilm formation.

### Proteomic analysis of statically cultured *X. citri* and *hrpB*^−^ strains

**Figure 5:**
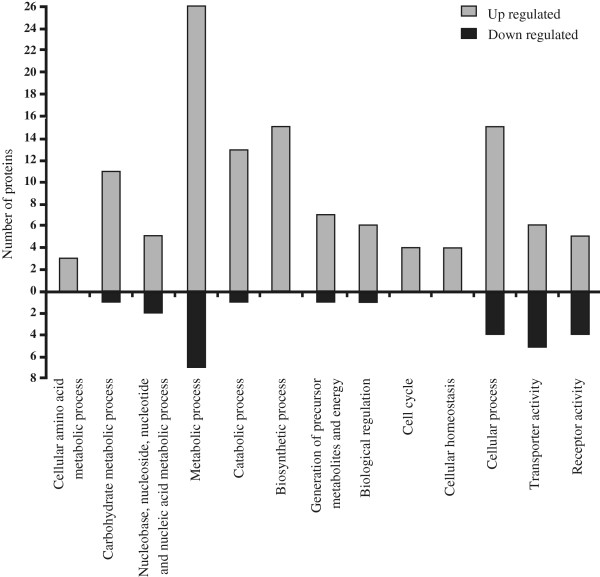
**Gene ontology (GO) terms enriched in differentially expressed proteins between *X. citri *and *hrpB***^**− **^**static cells.** Proteins up-regulated and down-regulated in the *hrpB*^−^ mutant relative to *X. citri* in the main enriched categories are shown. The GO enrichment analysis was performed using Blast2GO.

In order to gain new insights about the role of T3SS in biofilm formation, a proteomic analysis was performed to identify differentially expressed proteins between *X. citri* and the *hrpB*^*−*^ mutant grown statically. A total of 49 differentially expressed protein spots were detected of which 32 were up- and 17 down-regulated in the *hrpB*- mutant in comparison to *X. citri* (Table 1). Identified proteins were used to determine enriched GO categories in biological processes and molecular function. The main enriched categories for the up- and down-regulated proteins with an average fold change of minimum ± 1.5 and p value < 0.05 in the *hrpB*^−^ mutant relative to *X. citri* were represented graphically (Figure 5). The categories that showed a major enrichment in the up-regulated proteins in the *hrpB*- mutant include ‘metabolic process’, ‘catabolic process’, ‘biosynthetic process’ and ‘generation of precursor metabolites and energy’. Moreover, ‘cell cycle’, ‘cellular homeostasis’ and ‘cellular process’ were categories enriched in up-regulated proteins in this mutant. Most of the identified proteins in the categories of ‘transporter activity’ or ‘receptor activity’ belong to different classes of outer membrane proteins (OMPs) such as: FadL (XAC0019), that allows the passage of fatty acids [26], OmpW (XAC3664), involved in the transport of small hydrophilic molecules across the bacterial outer membrane [27] and RpfN (XAC2504), which was reported to play a role in carbohydrate transport [28]. In these categories also several TonB-dependent transporters (TBDTs), which are outer membrane transporters involved in the active uptake and/or in signal transduction [29], as well as two Oar (OmpA-related) proteins were detected as differentially expressed between the two strains.

**Table 1:** **Differentially expressed protein spots between *X. citri *** **and *hrpB***^**− **^**strains statically cultured in XVM2 with a change abundance of minimum 1.5 fold and p value of < 0.05 (ANOVA)**

***X. citri *** **gene no.**	**Protein name**	**MOWSE score**	**Accession no.**	**Predicted MW/p** ***I***	**Observed MW/p** ***I***	**Peptide match/coverage**	**Fold change compared to WT**
01 Metabolism							
01.01 Amino acid metabolism							
XAC0125	Aspartate/tyrosine/aromatic aminotransferase	350	Q8PR41_XANAC	43.3/5.72	49.0/4.8	19/38%	1.9
XAC4034	Shikimate 5-dehydrogenase	297	AROE_XANAC	29.9/4.93	30.0/5.9	19/17%	2.4
XAC2717	Tryptophan synthase subunit b	31	TRPB_XANAC	43.3/5.88	53.0/4.6	2/4%	7.5
XAC3709	Tryptophan repressor binding protein	48	Q8PGA8_XANAC	20.0/6.40	10.0/4.4	3/17%	−1.6
01.02 Nitrogen, sulfur and selenium metabolism							
XAC0554	NAD(PH) nitroreductase	208	Y554_XANAC	21.0/5.83	18.0/4.7	14/38%	4.6
01.03 Nucleotide/nucleoside/nucleobase metabolism							
XAC1716	CTP-synthase	125	PYRG_XANAC	61.7/5.91	67.0/4.5	14/21%	3.5
01.05 C-compounds and carbohydrate metabolism							
XAC2077	Succinate dehydrogenase flavoprotein subunit	192	Q8PKT5_XANAC	65.8/5.89	66.0/4.6	20/25%	2.2
XAC1006	Malate dehydrogenase	1054	MDH_XANAC	34.9/5.37	45.0/5.4	55/50%	−1.8
XAC3579	Phosphohexose mutases (XanA)	98	Q8PGN7_XANAC	49.1/5.29	54.0/5.6	7/10%	1.7
XAC3585	DTP-glucose 4,6-dehydratase	235	Q8PGN1_XANAC	38.6/5.86	48.0/4.7	12/17%	2.1
XAC0612	Cellulase	245	Q8PPS3_XANAC	51.6/5.76	57.0/4.9	23/32%	2.6
XAC3225	Transglycosylase	178	Q8PHM6_XANAC	46.2/5.89	53.0/4.8	14/22%	−1.6
01.06 Lipid, fatty acid and isoprenoid metabolism							
XAC3300	Putative esterase precursor (EstA)	96	Q8PHF7_XANAC	35.9/6.03	62.0/6.2	3/4%	−3.1
XAC1484	Short chain dehydrogenase precursor	104	Q8PME5_XANAC	26.0/5.97	30.0/4.4	5/9%	2.2
01.06.02 Membrane lipid metabolism							
XAC0019	Outer membrane protein (FadL)	167	Q8PRE4_XANAC	47.3/5.18	46.0/6.1	8/10%	−10.0
XAC0019	Outer membrane protein (FadL)	79	Q8PRE4_XANAC	47.3/5.18	35.0/6.0	7/13%	−6.2
01.20 Secondary metabolism							
XAC4109	Coproporphyrinogen III oxidase	46	HEM6_XANAC	34.6/5.81	37.0/4.9	8/19%	1.5
02 Energy							
02.01 Glycolysis and gluconeogenesis							
XAC1719	Enolase	90	ENO_XANAC	46.0/4.93	55.0/5.9	7/13%	1.7
XAC3352	Glyceraldehyde-3-phosphate dehydrogenase	267	Q8PHA7_XANAC	36.2/6.03	46.0/4.4	24/28%	2.6
XAC2292	UTP-glucose-1-phosphate uridylyltransferase (GalU)	92	Q8PK83_XANAC	32.3/5.45	38.0/5.3	13/30%	4.2
02.07 Pentose phosphate pathway							
XAC3372	Transketolase 1	85	Q8PH87_XANAC	72.7/5.64	69.0/4.9	5/7%	5.0
02.11 Electron transport and membrane-associated energy conservation							
XAC3587	Electron transfer flavoprotein a subunit	50	Q8PGM9_XANAC	31.8/4.90	34.0/5.5	6/14%	2.3
10 Cell cycle and DNA processing							
10.03 Cell cycle							
XAC1224	Cell division topological specificity factor (MinE)	33	MINE_XANAC	9.6/5.37	12.0/4.9	1/14%	2.7
10.03.03 Cytokinesis/septum formation and hydrolysis							
XAC1225	Septum site-determining protein (MinD)	143	Q8PN48_XANAC	28.9/5.32	34.0/5.6	19/26%	2.3
11 Transcription							
XAC0996	DNA-directed RNA polymerase subunit a	104	RPOA_XANAC	36.3/5.58	33.0/5.0	5/7%	−4.3
XAC0966	DNA-directed RNA polymerase subunit b	150	RPOC_XANAC	155.7/7.82	35.0/4.6	16/8%	−3.3
14 Protein fate (folding, modification and destination)							
14.01 Protein folding and stabilization							
XAC0542	60 kDa chaperonin (GroEL)	199	CH60_XANAC	57.1/5.05	41.0/5.5	15/27%	−11.2
16 Protein with binding function or cofactor requirement							
XAC2726	Adenine-specific methylase (Dam methylase)	84	Q8PJ19_XANAC	33.9/4.70	41.0/6.2	10/12%	−1.9
XAC1362	GTN reductase	44	Q8PMR4_XANAC	39.4/5.37	50.0/5.3	7/10%	2.3
XAC3664	OmpW family outer membrane protein precursor	226	Q8PN48_XANAC	23.8/4.97	28.0/6.2	12/13%	2.3
30 Cellular communication/Signal transduction mechanism							
XAC0291	Oar protein ( TonB-dependent transporter)	50	Q8PQN2_XANAC	107.9/5.29	108.0/5.7	2/1%	4.3
XAC2672	Oar protein ( TonB-dependent transporter)	280	Q8PJ70_XANAC	117.4/5.10	90.0/5.9	19/18%	2.4
XAC4273	TonB-dependent transporter	100	Q8PJL0_XANAC	109.2/5.14	90.0/5.6	3/3%	2.8
XAC1143	TonB-dependent transporter	576	Q8PND0_XANAC	87.7/5.21	70.0/6.1	30/33%	1.7
XAC3050	TonB-dependent transporter	596	Q8PI48_XANAC	105.8/4.76	64.0/6.2	30/16%	−3.0
XAC3444	TonB-dependent transporter	1280	Q8PH16_XANAC	103.2/4.79	90.0/6.3	84/37%	3.9
XAC3168^*^	TonB-dependent transporter	98	Q8PHT1_XANAC	87.3/5.20	59.0/6.0	3/3%	−3.1
XAC3166^*^	TonB-dependent transporter	410	Q8PHT3_XANAC	84.5/4.95	69.0/6.1	22/18%	−2.9
XAC3489	TonB-dependent transporter	685	Q8PGX3_XANAC	88.9/4.93	69.0/5.9	40/24%	−1.7
XAC1413	Outer membrane protein assembly factor BamA	135	Q8PML3_XANAC	87.6/5.53	88.0/5.4	13/15%	2.8
32 Cell rescue, defense and virulence							
XAC2504^*^	Regulator of pathogenicity factors (RpfN)	271	Q8PJM6_XANAC	41.3/5.98	49.0/4.4	21/16%	−4.8
XAC0907	Alkyl hydroperoxide reductase subunit C	240	O06464_XANAC	20.6/6.15	20.0/4.2	28/61%	1.3
32.07 Cellular detoxification							
XAC1474	Glutathione transferase	39	Q8PMF5_XANAC	23.9/6.06	22.0/4.7	4/8%	1.7
34 Interaction with the environment							
34.01 Homeostasis							
XAC1149	Bacterioferritin	100	Q8PNC4_XANAC	21.2/4.71	20.0/6.3	6/20%	2.1
XAC0493	Bacterioferritin	152	Q8PQ38_XANAC	18.3/4.80	12.0/6.5	19/43%	2.5
XAC1533	Dihydrolipoamide dehydrogenase	336	Q8PM99_XANAC	50.5/5.80	59.0/4.6	34/47%	4.0
42 Biogenesis of cellular components							
XAC1230	Putative membrane protein	71	Q8PN43_XANAC	43.1/6.88	24.0/4.4	4/11%	−3.5
99 Unclassified proteins							
XAC1262	Protein of unknown function (Aminopeptidase)	121	Q8PN12_XANAC	63.4/5.85	68.0/4.6	13/15%	5.3
XAC1344	Protein of unknown function (CcmA)	67	Q8PMT2_XANAC	18.7/5.45	23.0/5.7	4/18%	−1.7

*Protein spots 240 and 398 were previously named “ferric enterobactin receptor” are now classified as TonB-dependent transporter, while protein spot 31 previously identified as “carbohydrate selective porin” and is now classified as Regulator of pathogenicity factors.

### The lack a T3SS enhances *X. citri* EPS production and decreases bacterial motility

**Figure 6:**
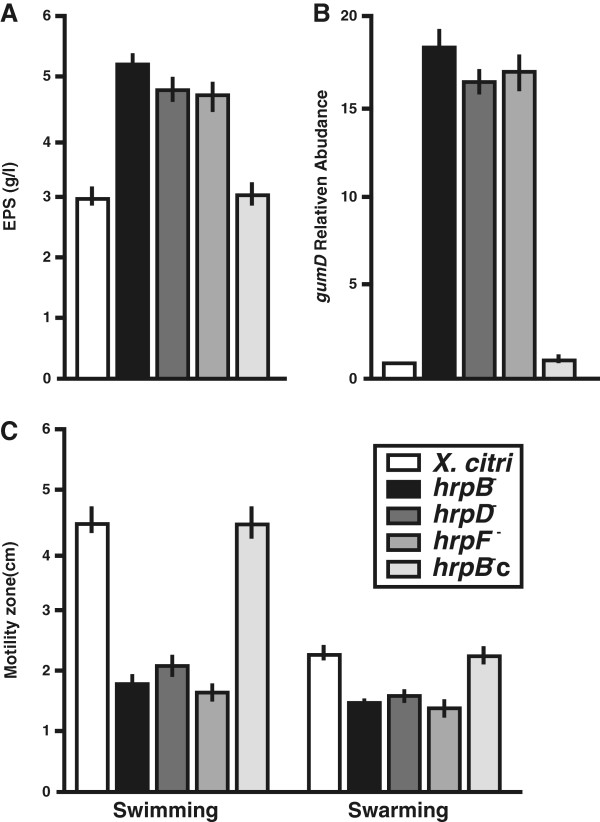
**EPS production and bacterial motility assays in *X. citri*, the *hrp *mutants and the *hrpB***^**−**^**c strains. (A)** Quantification of EPS present in the supernatant fraction of cultures of the different strains. Quadruplicate measurements were made for each strain and an average of all measurements was obtained. Error bars indicate standard deviations. **(B)** RT-qPCR assay to determine *gumD* expression of the different stains relative to *X. citri*. Values are the means of four biological replicates with three technical replicates each. **(C)** Quantification of bacterial swimming and swarming motility. Results are the average of the motility zones of 16 Petri dishes per strain. Error bars indicate the standard deviation.

The proteomic assay detected an over-expression of the enzymes XanA and GalU in the *hrpB*^−^ mutant compared to *X. citri* (Table 1). The XanA enzyme encodes a bifunctional phosphoglucomutase/phosphomannomutase involved in the synthesis of both glucose 1-phosphate and mannose 1-phosphate. Glucose 1-phosphate is then converted to UDP-glucose by GalU and mannose 1-phosphate to GDP-mannose by mannose 1-phosphate guanylyltransferase. These nucleotide sugars are directly implicated in EPS synthesis [30,31]. Production of EPS was measured in *X. citri*, the *hrp* mutants and the *hrpB*^−^c strains and results showed that EPS production in these mutants was over 1.7 times that in *X. citri* and *hrpB*^−^c strain (p < 0.05) (Figure 6A). Additionally, the expression of *gumD*, a gene encoding a protein of the EPS biosynthetic pathway, was analyzed by RT-qPCR in all the strains. The results showed that the transcript levels of *gumD* were over 17 times higher in *hrp* mutant strains as compared to *X. citri* and the *hrpB*^−^c strain (p < 0.05) (Figure 6B). Moreover, the proteomic analysis also showed a down-regulation of the outer membrane protein XAC0019 in the *hrpB*^−^ mutant (Table 1) and recently, it has been shown that this protein is necessary for *X. citri* swimming [32]. Furthermore, CcmA that is required for bacterial motility [33,34] was also down-regulated in the *hrpB*^−^ mutant (Table 1). Therefore, bacterial motility was assayed for the *hrp* mutants and results showed that *X. citri* and the *hrpB*^−^c strain moved about 2.5 and 1.25 further in swimming and swarming plates respectively, than the *hrp* mutants (p < 0.05) (Figure 6C) (Additional file 2: Figure S2).

## Discussion

The role of T3SS in bacterial pathogenesis as a machine involved in effector protein delivery is well established, however, little is known about other functions in bacterial behavior that this system may have. Given that biofilm formation is required for *X. citri* to achieve full virulence, we used *X. citri* as a model to gain further insights into the functional role of T3SS in biofilm formation. By comparing the capacity of biofilm formation of three T3SS mutants and *X. citri* and also performing a proteomic assay with the *hrpB*^*−*^ mutant, which revealed differentially expressed proteins between both strains, we demonstrated that T3SS is involved in biofilm formation in *X. citri*.

To date the involvement of *X. citri* T3SS in bacterial attachment and survival on leaf tissue has not been studied. In this work we observed that the adherence of different T3SS mutants to host cell tissue was not altered. Studies in several pathogenic bacteria, such as *Salmonella typhimurium*[35], *E. coli*[36,37] and the plant pathogen *P. syringae*[38] revealed that mutants unable to produce T3SS appendages become affected in their interactions with host cells. However, in the phytopathogen *Ralstonia solanacearum*, it has been shown that the lack of a T3SS pilus does not affect attachment to plant cells [39], and this is consistent with our observation that adherence of *X. citri* to the host tissue was not affected by the absence of a functional T3SS. In addition, we determined that T3SS is required for *X. citri* survival on citrus leaves and that T3SS genes are expressed while bacteria reside on the plant surface. Expression of T3SS genes on the leaf surface was also detected in *Xanthomonas euvesicatoria* cells suggesting a role for T3SS in epiphytic survival of the bacteria [40]. In a recent report, it was revealed that the survival of *Pseudomonas syringae* T3SS-deficient strains on leaf surfaces is reduced, supporting a role of T3SS and effector proteins in the promotion of epiphytic bacterial survival [41]. Our results suggest that T3SS plays a role in *X. citri* leaf-associated survival on the leaf surface by enabling biofilm formation.

The proteomic study revealed differentially expressed proteins between *X. citri* and the *hrpB*^*−*^ mutant strain and GO analysis detected enrichment of up-regulated proteins in different metabolic processes and generation of energy in the *hrpB*^−^ mutant. Similarly, in a previous proteomic study, these categories were also enriched with up-regulated proteins in *X. citri* planktonic cells compared to biofilm, suggesting a slower metabolism and reduction in aerobic respiration in the *X. citri* biofilm [42]. Therefore, the higher expression of proteins involved in these processes in the *hrpB*^−^ mutant compared to *X. citri* may be caused by the lack of biofilm formation of the mutant.

It is remarkable that among the differentially expressed proteins between the mutant and the wild type strain, some have been previously characterized as involved in biofilm formation in *X. citri* or in other pathogenic bacteria. Such is the case of DNA-directed RNA polymerase subunit β [32], tryptophan synthase [43], GroEL [44,45], FadL [32,42,46] and several TBDTs [42,47]. Interestingly, high intracellular L-tryptophan concentration prevents biofilm formation and triggers degradation of mature biofilm in *E. coli*[43]. The proteomic assay showed that tryptophan synthase (XAC2717) was up-regulated, while the tryptophan repressor binding protein (XAC3709) was down-regulated in *hrpB*^−^ strain suggesting a link also between tryptophan metabolism and biofilm formation in *X. citri*. Another example is the outer membrane protein XAC0019 that displays high homology to the fatty acid transport porin FadL. *Pseudomonas fluorescens* mutants in the *fadL* gene showed defects in their ability to develop a biofilm on a abiotic surfaces leading to the suggestion that long chain fatty acids bind to FadL thereby altering surface hydrophobicity, and adhesion characteristics [46]. Consistent with this, a recent work showed that a *X. citri* mutant in XAC0019 displays reduced capacity to form a biofilm [32] and its expression is increased during *X. citri* biofilm formation [42]. In the present study, XAC0019 protein was down-regulated in the *hrpB*^−^ mutant impaired in biofilm formation, reinforcing the role of this protein in this process.

Enzymes involved in EPS production XanA and GalU, [30,31] were up-regulated in the *hrpB*^−^ mutant. Consistently, all the *hrp* mutant analyzed in this work produced larger amounts of EPS in comparison with *X. citri* and also had higher expression levels of *gumD*. Recent reports have shown that *X. citri galU* mutant strain is not pathogenic and also loses its capacity to form a biofilm due to a reduction in EPS production [30,32], and that a *X. citri xanA* mutant has an altered capacity for biofilm formation [47]. Although, the *hrp* mutants are impaired in biofilm formation, these mutants produce more EPS than *X. citri*. This interesting result open new hypotheses about the link between T3SS and EPS production, thus further studies are needed to unravel this issue. In other pathogens, such as *P. aeruginosa*, T3SS gene expression is coordinated with many other cellular activities including motility, mucoidy, polysaccharide production, and also biofilm formation [48].

Bacterial motility was impaired in the *hrp* mutants and consistently, proteins known as involved in these processes such as the outer membrane protein XAC0019 [32] and the bactofilin CcmA [33,34] were down-regulated in the *hrpB*^−^ mutant. Besides, swarming motility was less affected than swimming in the *hrp* mutants compared with *X. citri*. This may be due to the fact that in *X. citri* swarming motility depends on flagella and also on the amount of EPS secreted [16], and since these mutants over-produced EPS swarming was less affected than swimming.

This work demonstrated that in *X. citri* T3SS is involved in multicellular processes such as motility and biofilm formation. Furthermore, our results suggest that T3SS may also have an important role in modulating adaptive changes in the cell, and this is supported by the altered protein expression when this secretion system is not present. It was previously shown that an *E. coli* O157 strain mutant in the additional T3SS named ETT2 is impaired in biofilm formation [13]. It was also suggested that deletion of ETT2 might cause structural alterations of the membrane modifying bacterial surface properties, thus affecting bacteria-bacteria interactions or the interaction with host cells [13]. Further, it was proposed that these structural alterations could trigger a signal that activates differential gene expression and/or protein secretion [13]. In line with this, we propose that in *X. citri* the ‘Hrp pilus’ structure *per se*, or its interaction with a solid surface, stabilizes the outer membrane structure, hence the lack of T3SS may trigger membrane remodeling itself. These membrane modifications in turn may change the pattern of protein expression, leading to the impairment of cellular processes directly related to bacterial virulence including biofilm formation. Another possibility is that the ‘Hrp pilus’ may function like an attachment device or flagellum. Future studies are likely to add further insights into the exact role and modes of operation of *X. citri* ‘Hrp pilus’ in biofilm formation and motility.

## Conclusions

This work demonstrates that the presence of T3SS in *X. citri*, besides its participation in the secretion of effector proteins is also required for biofilm formation, motility and survival on leaf tissue revealing novel functions for this secretion system in *X. citri*. In biofilm formation, T3SS may have an important role in modulating adaptive changes that lead to this process. Some of these changes are revealed by variations in proteins involved in metabolic processes, energy generation, EPS production and bacterial motility as well as in outer membrane proteins between the wild type strain and the T3SS mutant. In summary, the present study reveals novel contributions of this protein secretion system to bacterial virulence.

## Methods

### Bacterial strains, culture conditions and media

*X. citri* strain Xac99-1330 was isolated from *C. sinensis* and kindly provided by Blanca I. Canteros (INTA Bella Vista, Argentina). The *hrpB*^−^ mutant was constructed in previous work [19]. Here, *hrpB*^−^c complemented strain was constructed by cloning the region from *hrpB5* to *hrcT* in the replicative plasmid pBBR1MCS-5 [20] under the control of the *lacZ* promoter. This region was amplified from *X. citri* genomic DNA with the oligonucleotides: HrpB5F-Hind (5′ ATAGAAGCTTCATGCGTCTCTGGTTGAGGTC 3′) and HrcTR-Bam (5′ ATCAGGATCCTCAGTGCGACGCGGCTCTCT 3′) and cloned into pBBR1MCS-5 previously digested with the restriction enzymes *Hind*III and *Bam*HI. The resulting construction was electroporated into the *hrpB*^−^ strain and the complemented mutant strain was selected by for gentamicin antibiotic resistance. For confocal laser scanning microscopy analyses, a GFP-expressing *hrpB*^*−*^ strain was obtained. To this end, the coding sequence for EGFP from the broad-host-range vector pBBR1MCS-2EGFP [16] was digested with *BamH*I and *Xba*I and ligated in frame with the LacZ-α-peptide of the pBBR1MCS-5 vector [20] previously digested with the same enzymes, rendering the plasmid pBBR1MCS-5EGFP. *E. coli* S17-1 cells transformed with this plasmid were conjugated with the *hrpB*^−^ strain and the cells carrying the plasmid pBBR1MCS-5EGFP were selected for Gm resistance. All strains were grown at 28°C in Silva Buddenhagen (SB) medium [16] or in XVM2 medium [49]. Antibiotics were used at the following final concentrations: 25 μg/ml ampicillin (Amp), 5 μg/ml gentamicin (Gm) and 40 μg/ml kanamycin (Km).

### Plant material

Orange (*Citrus sinensis* cv. Valencia) was used as the host plant for *X. citri*. All plants were grown in a growth chamber with incandescent light at 28°C with a photoperiod of 16 h.

### Biofilm assays

For biofilms development, bacteria were grown in SB with shaking until exponential growth phase and then diluted 1:10 in fresh XVM2 medium containing appropriate antibiotics. A 2 ml aliquot of diluted bacterial suspension was placed in borosilicate glass tubes or in 24-well PVC plates and incubated statically for seven days at 28°C. The quantification of biofilm formation by CV staining was carried out as previously described [50]. Briefly, the culture medium was decanted and the absorbance of planktonic cells was measured at 600 nm using a UV-visible spectrophotometer (Synergy 2 Reader, BioTek). After washing the tubes three times with distilled water (dH_2_O) during 10 min with gentle agitation, the remaining attached cells were incubated for 10 min at 60°C and stained with 0.1% (w/v) CV for 30 min at room temperature. Excess CV stain was removed by washing under running tap water. The CV stain was solubilized by the addition of 1.5 ml ethanol:acetone (80:20, v/v) to each tube and quantified by measuring the absorbance at 600 nm. The relative absorbance (Relative abs.) was calculated as: CV Abs. 600 nm/Planktonic cells Abs. 600 nm. Values represent the mean from seven tubes for each strain, data were statistically analyzed using one-way analysis of variance (ANOVA) (p < 0.05).

### Confocal analysis of biofilm architecture

*In vitro* biofilm of the GFP-expressing *hrpB*^−^ mutant and *X. citri* previously constructed [16] grown in 24-well PVC plates in XVM2 medium were analyzed after seven days by confocal laser scanning microscopy (Nikon Eclipse TE-2000-E2). For biofilms assays on leaf surfaces, overnight cultures of both GFP-expressing strains grown in XVM2 medium were centrifuged, washed and resuspended in phosphate buffer (pH 7.0) to the same OD_600_ and 20 μl of each bacterial suspension were applied on abaxial leaf surfaces. These biofilms were also analyzed after seven days by confocal laser scanning microscopy (Nikon Eclipse TE-2000-E2).

### Adhesion assays

The adhesion capacity to leaf surfaces was measured as described previously [16]. Overnight cultures of the different strains in XVM2 medium were centrifuged to recover cell pellets, washed and resuspended in phosphate buffer (pH 7.0) to the same optic density measured at 600 nm (OD600). Then, 20 μl of each bacteria suspension were place on abaxial leaf surfaces and incubated for 6 h at 28°C in a humidified chamber. After washing the non-adhered cells, bacteria were stained with CV, the CV stain was extracted from the bacterial drops with 95% (v/v) ethanol by pipetting up and down with a 20 μl micropipette. Quantification of the extracted CV stain was carried out by measuring the absorbance at 590 nm as described above. For each strain 20 stained drops were quantified and data were statistically analyzed using one-way ANOVA (p < 0.05).

### Quantification of leaf-associated survival

Leaf-associated fitness was evaluated as previously described [51]. Briefly, overnight cultures in SB medium were centrifuged to recover bacteria cell pellets, washed and resuspended in 10 mM phosphate buffer (pH 7.0) at a concentration of 10^9^ CFU/ml. These bacterial suspensions were sprayed onto leaves until each leaf surfaces were uniformly covered. Old citrus leaves were used since the greater thickness of the cuticles of these leaves naturally render the leaves resistant to bacterial entry (unpublished results). Four different leaves were inoculated with each strain, leaves were photographed and the surfaces were quantified using the software Image-Pro (Media Cybernetics). Leaves were collected on different days post-inoculation and transferred to borosilicate glass flasks containing 10 mM potassium phosphate buffer (pH 7.0). Flasks were submerged in a sonicator (Branson model #5510) for 10 min. Subsequently, each flask was vortexed for 5 sec, bacteria were recovered by centrifugation and serial dilutions were plated on SB plates containing Ap to count bacterial colonies. Results were expressed in CFU/cm^2^ of inoculated leaves. Values represent an average of four leaves assayed for each strain, the data were statistically analyzed using one-way ANOVA (p < 0.05).

### RNA preparation and RT-qPCR

Total RNA from bacterial cultures grown at the indicated conditions and from bacteria recovered from leaves at the indicated times were isolated using TRIzol® reagent (Invitrogen), according to the manufacturer’s instructions. The RT-qPCRs were performed as previously described [52] with the specific oligonucleotides detailed in Additional file 3: Table S1. As a reference gene, a fragment of 16S rRNA (XAC3896) was amplified using the same RT-qPCR conditions. To control that no bacterial DNA contamination was present in the samples, the same PCR reactions were carried out without retrotranscription and non amplification was observed. To ascertain the absence of plant RNA in bacterial samples controls with plant actin primers were carried out (data not shown). Values were normalized by the internal reference (Ct_r_) according to the equation ΔCt = Ct – Ct_r_, and quantified as 2^–ΔCt^. A second normalization using a control (time = 0 days) (Ct_c_), ΔΔCt = Ct – Ct_c_, producing a relative quantification: 2^–ΔΔCt^[53]. Values are the means of four biological replicates with three technical replicates each. Results were analyzed by Student *t*-test (p < 0.05) and one-way ANOVA (p < 0.05).

### Protein extraction and resolubilization for the proteomic analysis

Biofilms of statically grown bacterial cultures were obtained as previously described [42]. After seven days of static growth, the XVM2 medium was carefully removed and biofilms were collected by pipetting, transferred to a new tube and pelleted by centrifugation prior to protein extraction. Biofilm proteins were extracted and resuspended in urea buffer (9 M urea, 2 M thiourea and 4% (w/v) 3-[(3-cholamidopropyl)dimethylammonio]-1-propanesulfonate (CHAPS)) with vigorous vortexing at room temperature. Concentration of total protein extracts was estimated using a modified Bradford assay [54] and using bovine serum albumin as standard. Protein extracts were prepared from three biological replicates for each strain.

### Proteomic analyses

Total proteins from biofilm cells were extracted and labeled using the fluorescent cyanine three-dye strategy (CyDyes; GE Healthcare), as described in [42]. *X. citri* and *hrpB*^−^ protein samples were labeled with Cy3 and Cy5, respectively, according to manufacturer’s instructions. Protein extractions were performed from three independent biological samples, and two technical replicate gels for each experiment were run. Protein separation, quantification by two-dimensional-difference in-gel electrophoresis (2D-DIGE), comparative analysis and protein identification were also carried out as previously described [42]. Normalized expression profile data were used to statistically assess changes in protein spot expression. Differentially expressed protein spots between the two groups were calculated using the Student *t*-test with a critical p-value ≤ 0.05 and the permutation-based method to avoid biased results that may arise within replicate gels if spot quantities are not normally distributed. The adjusted Bonferroni correction was applied for false discovery rate (FDR) to control the proportion of false positives in the result set. Principal component analysis was performed to determine samples and spots that contributed most to the variance and their relatedness. Protein spots with a minimum of 1.5 fold change and p values < 0.05 only were considered as significantly differentially expressed between the two strains.

### Quantification of EPS production

Quantification of EPS production was performed as previously described [55]. Briefly, bacterial strains were cultured to the stationary growth phase in 50 ml of SB liquid medium supplemented with 1% (w/v) glucose in 250 ml flasks, using an orbital rotating shaker at 200 rpm at 28°C. Cells were removed by centrifugation at 2,500 × g for 30 min at room temperature, and the supernatant fluids were separately supplemented with KCl at 1% (w/v) and 2 volumes of 96% (v/v) ethanol and then incubated for 30 min at -20°C to promote EPS precipitation. Precipitated crude EPS were collected, dried and weighed. Results were expressed in grams per culture liter. Quadruplicate measurements were made for each strain and an average of all measurements was obtained, data were statistically analyzed using one-way ANOVA (p < 0.05).

### Swimming and swarming assays

Swimming and swarming motility were measured as previously described [16]. The SB plates fortified with 0.3% (w/v) or 0.7% (w/v) agar respectively were centrally inoculated with 5 μl of 1 × 10^7^ CFU/ml cultures in exponential growth phase. Inoculated Petri dishes were then incubated in a humidity chamber for two days at 28°C and the motility zones were measured. Results are the average of the motility zones of sixteen Petri dishes per strain. Data was statistically analyzed using one-way ANOVA (p < 0.05).

## Competing interests

The authors declare that they have no competing interests.

## Authors’ contributions

JO and NG conceived the project and designed the experiments. TZ, LT, CM, GGS, CGG, FAF and NG designed and performed the experiments. All authors contributed to the analysis and interpretation of the data and LT, CM, CG, JO and NG wrote the manuscript. All authors read and approved the manuscript.

## Supplementary Material

Additional file 1: Figure S1 Characterization of the *hrpB*^*−*^ complemented strain on HR and pathogenicity. (A) Schematic organization of the *hrp* cluster of *X. citri* that was constructed based on the *X. citri subsp. citri* strain 306 genome sequence [1]. Boxes correspond to ORFs, arrows indicate orientation of the *hrp* operons. The *hrp*, *hpa* and *hrc* genes are indicated. Dotted boxes indicated the genomic regions replaced by mutagenesis. Bellow of the scheme, the black box represents the genomic fragment cloned in pBBR1MCS-5 to complement the *hrpB*^*−*^ mutant strain. (B) Bacterial suspensions of *X. citri*, the *hrpB*^*−*^ mutant and the *hrpB*^*−*^c strains were inoculated at 10^8^ CFU/ml into the intercellular spaces of fully expanded tomato, cotton and pepper leaves. A representative photograph of a leaf is shown after 1 day of inoculation. (C) As in B, bacterial suspensions at 10^7^ CFU/ml were inoculated into the intercellular spaces of fully expanded citrus leaves. A representative photograph of a leaf is shown after 8 days of inoculation. (D) RT-qPCR to determine *CsLOB1* expression levels in leaves after 48 hours of infection with *X. citri*, the *hrpB*^*−*^ mutant and *hrpB*^*−*^c strain. Bars indicate the expression levels relative to buffer infiltrations. Values are the means of four biological replicates with three technical replicates each. Click here for file

Additional file 2: Figure S2 Swimming and swarming assays. Representative photographs of Petri dishes with *X. citri*, the *hrp* mutants and the *hrpB*^*−*^c strain after 2 days of inoculation. Scale bars: 10 mm. Click here for file

Additional file 3: Table S1 Oligonucleotides used in RT-qPCR assays. Click here for file
