# Supplementary figures and images for: The type III protein secretion system contributes to Xanthomonas citri subsp. citri biofilm formation
Source: BMC Microbiol. 2014 Apr 18;14:96. doi: 10.1186/1471-2180-14-96 (PMC4021560; doi:10.1186/1471-2180-14-96)

**A**

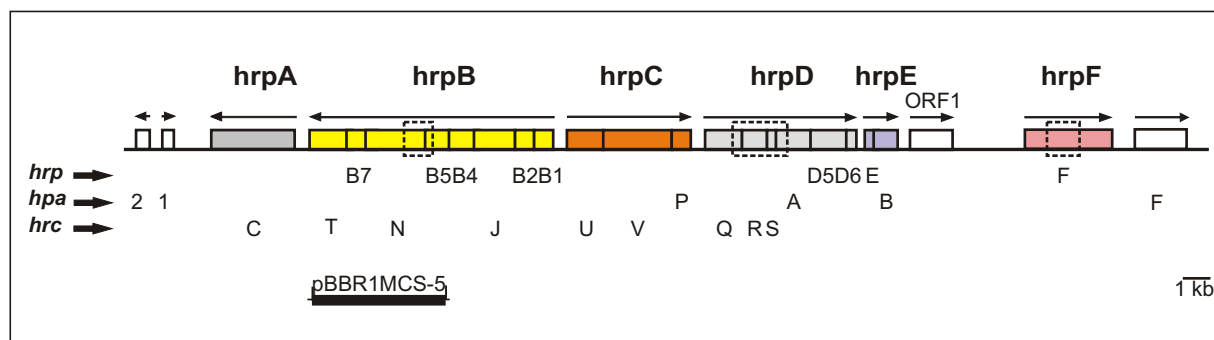

**B**

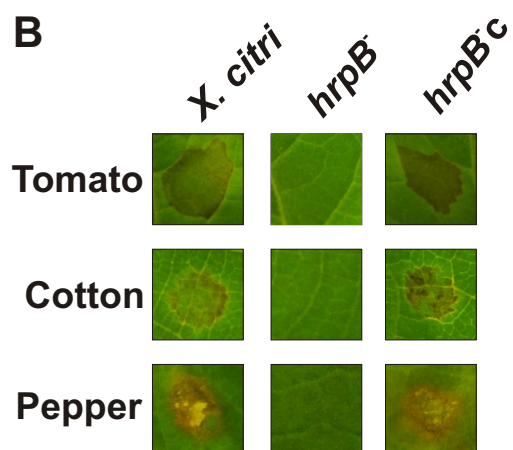

**C**

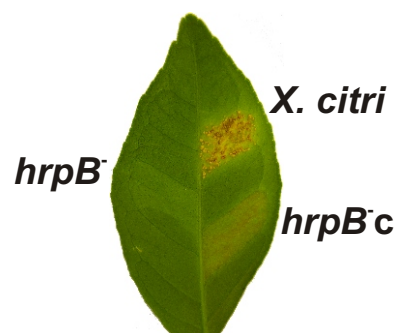

**D**

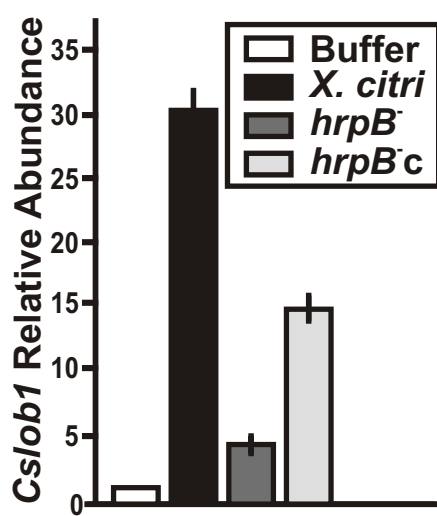

Supplement: Additional file 1: Figure S1 — Characterization of the hrpB− complemented strain on HR and pathogenicity. (A) Schematic organization of the hrp cluster of X. citri that was constructed based on the X. citri subsp. citri strain 306 genome sequence [1]. Boxes correspond to ORFs, arrows indicate orientation of the hrp operons. The hrp, hpa and hrc genes are indicated. Dotted boxes indicated the genomic regions replaced by mutagenesis. Bellow of the scheme, the black box represents the genomic fragment cloned in pBBR1MCS-5 to complement the hrpB− mutant strain. (B) Bacterial suspensions of X. citri, the hrpB− mutant and the hrpB−c strains were inoculated at 108 CFU/ml into the intercellular spaces of fully expanded tomato, cotton and pepper leaves. A representative photograph of a leaf is shown after 1 day of inoculation. (C) As in B, bacterial suspensions at 107 CFU/ml were inoculated into the intercellular spaces of fully expanded citrus leaves. A representative photograph of a leaf is shown after 8 days of inoculation. (D) RT-qPCR to determine CsLOB1 expression levels in leaves after 48 hours of infection with X. citri, the hrpB− mutant and hrpB−c strain. Bars indicate the expression levels relative to buffer infiltrations. Values are the means of four biological replicates with three technical replicates each. [file 1471-2180-14-96-S1.pdf]

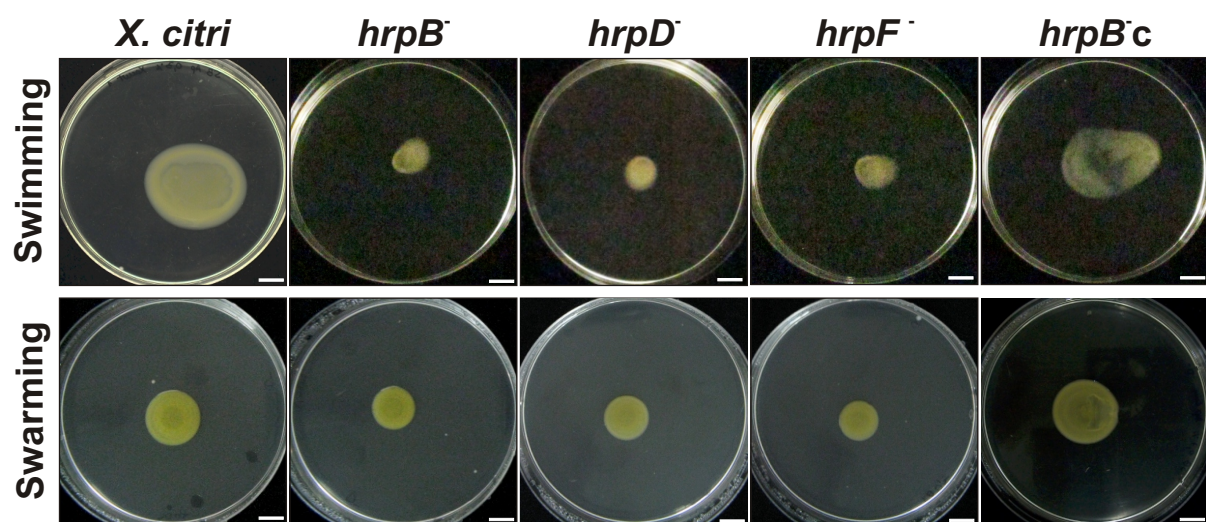

Supplement: Additional file 2: Figure S2 — Swimming and swarming assays. Representative photographs of Petri dishes with X. citri, the hrp mutants and the hrpB−c strain after 2 days of inoculation. Scale bars: 10 mm. [file 1471-2180-14-96-S2.pdf]
