# Supplementary material for: The type III protein secretion system contributes to Xanthomonas citri subsp. citri biofilm formation
Source: BMC Microbiol. 2014 Apr 18;14:96. doi: 10.1186/1471-2180-14-96 (PMC4021560; doi:10.1186/1471-2180-14-96)
Supplement: Additional file 3: Table S1 — Oligonucleotides used in RT-qPCR assays. [file 1471-2180-14-96-S3.pdf]

Supplementary Table 1. Oligonucleotides used in RT-qPCR assays.

Gene name	Forward Oligonucleotide	Reverse Oligonucleotide
hrpG	ACAACATTCTGGCCTGGTAT	TTGTAGATGTGCTGCTCCAT
hrpX	CGATGATGAGGTCAGTTTGT	ACTGCGCAAAGCAATTCAAC
hrpE	ATCAGGATCCATGGAATTATTACCGC AAATCAG	ATACAAGCTTTTACTGGCCAACGAGCT GCTT
gumD	GCGCGGCCGTGGGATTGCTGAGT	TGGCGGCGCTGACGGAAGAACAC
16S	TGGTAGTCCACGCCCTAAACG	CTGGAAAGTTCCGTGGATGTC
CsLOB1	TCCACCAACCGAACCATACA	GGCACTTGCTTCATAGACCAT
Actin	TCAATTGGATACTTCAAAGTCAAAAT	ACGTGAATTCTAGTGTTTCGATAAGT
